# Phenolic Compounds Extracted from Cherry Tree (*Prunus avium)* Branches: Impact of the Process on Cosmetic Properties

**DOI:** 10.3390/antiox11050813

**Published:** 2022-04-22

**Authors:** Gaëlle Willig, Fanny Brunissen, Fanny Brunois, Blandine Godon, Christian Magro, Charles Monteux, Cédric Peyrot, Irina Ioannou

**Affiliations:** 1 URD Agro-Biotechnologies Industrielles (ABI), CEBB, AgroParisTech, 51110 Pomacle, France; gaelle.willig@agroparistech.fr (G.W.); fanny.brunissen@agroparistech.fr (F.B.); fanny.brunois@agroparistech.fr (F.B.); blandine.godon@agroparistech.fr (B.G.); 2 Chestnut, 26 Rue Barthélémy de Laffemas, 26000 Valence, France; christian.magro@chestnut-co.com (C.M.); charles.monteux@chestnut-co.com (C.M.)

**Keywords:** cherry tree branches, solvent extraction, phenolic compounds, catechin, prunin, bioactivities, antioxidant, anti-tyrosinase, antimicrobial, whitening agent

## Abstract

Cherry tree branches (*Prunus avium* var *burlat* Rosaceae) are agricultural by-products that are often neglected, yet they are rich in phenolic compounds and highly appreciated for their numerous biological activities. Extracts of cherry tree branches were evaluated for their use in cosmetics, particularly for their antioxidant, anti-tyrosinase, and antimicrobial activities. Samples were obtained by accelerated solvent extraction (ASE) at different ethanol percentages and different temperatures. Fourteen phenolic compounds were identified in the extracts by mass spectrometry. Three major compounds were identified (catechin, genistin, and prunin) representing 84 wt% of the total phenolic compounds. Optimal operating conditions maximizing the content of phenolic compounds were determined using a one factor at a time (OFAT) approach (70% aqueous ethanol, 70 °C). The extract obtained under these conditions also showed the highest antioxidant and anti-tyrosinase activities, certainly due to a high catechin content. Although the antimicrobial activities of extracts are less versatile than those of synthetic molecules, they are nonetheless interesting. According to these results, the extracts of cherry tree branches could be used in cosmetics for their interesting properties.

## 1. Introduction

Faced with strong consumer demand for natural products, the cosmetic industry has turned to bio-based molecules or plant extracts obtained from renewable and traceable materials. From a marketing point of view, some sources of active ingredients appear more attractive than others. This is particularly the case for flowers or fruits, especially the sweetest ones such as pomegranate, apple, coconut, or cherry. In addition, Japan–where the cherry tree is an important part of the local culture–has always used cherry leaves, stems, or stones in infusion or decoction in traditional medicine for their anti-inflammatory effect [1]. On this basis, numerous studies have been carried out to identify the molecules present in these extracts and to evaluate their potential cosmetic properties. The interest then quickly focused on the fruit itself, which is easily accessible and naturally rich in bioactive compounds such as anthocyanins, flavonoids, and phenolic acids [2]. The presence of these secondary metabolites confers to the extract numerous biological activities of cosmetic interest, such as anti-inflammatory [1], anti-aging [3,4], preservative [5], whitening agent [6], and many others [7,8]. However, the use of food resources to produce cosmetic ingredients quickly posed an ethical issue due to its competition with the food market. Thus, research turned to the by-products of the cherry food industry: downgraded fruits or fruit pomace from pressing, cherry stones, and stems [9]. Numerous studies have been performed to identify the bioactive compounds present in these biomasses [2,8]. For example, Afonso et al. realized a conventional extraction by maceration of cherry stones and stems to demonstrate the presence of saponin and phenolic compounds (quercetin, epicatechin, ellagic acid, chlorogenic acid, and catechin) in abundance in all extracts [10]. These by-products therefore also appear as an original source of active molecules for the cosmetic industries. Until recently, the arboricultural aspect of the crop was put aside by focusing on the fruit and its by-products, but every year the cherry trees are pruned to control their growth and favorize new sprouts. This annual pruning generates a large quantity of woody by-products (branches) that are not yet or hardly valued. In most cases, these are left on the ground or used for energy production (methanization). However, it has been shown that these branches (*Prunus avium* var *burlat* Rosaceae) are rich in phenolic compounds (PCs) [11]. Thus, it is possible, as for downgraded fruits, stones, and stems, to valorize them for their cosmetic activities. Few studies have focused on the extraction of secondary metabolites from branches. The most used process is conventional solvent extraction [11,12]. This extraction is carried out at room temperature, with a mixture of water/methanol mixture (80/20) or water/acetone (20/80), and with a liquid/solid ratio of 0.05 to 0.2 mL/mg. Intensified technologies, such as accelerated solvent extraction (ASE) [13], CO_2_-Sc extraction [13], and subcritical water extraction [14] have also been used. However, no optimization studies have been performed on the extraction of PCs from cherry tree branches to improve bioactivities sought after by the cosmetic industry. The objective of this work was to investigate the effect of the operating conditions of the extraction process on the content of different individual phenolic compounds (IPCs) as well as their cosmetic activities. For this purpose, the composition of PCs of a cherry branch extract was determined by mass spectrometry. Then, in a second part, the impact of the extraction temperature and the ethanol concentration of the extraction solvent was studied. Finally, the conditions for obtaining the most active extracts (anti-oxidant, anti-tyrosinase, and antimicrobial) were defined.

## 2. Materials and Methods

Catechin, isoquercetin, procyanidine B2, quercetin, tyrosinase from mushroom and 2,2-di(4-*tert*-octylphenyl)-1-picrylhydrazyl (DPPH), Folin-Ciocalteau reagent, (±)-6-hydroxy-2,5,7,8-tetra-methylchromate-2-carboxylic acid (Trolox), ethyl paraben, phenoxyethanol, and gallic acid were purchased from Merck. Genistein and kojic acid were ordered from TCI (Paris, France). Rutin, apigenin, chlorogenic acid, chrysin, and all solvents were purchased from Fischer Scientific (Illkirch, France). Neochlorogenic acid, epicatechin, genistin, and prunin were purchased from Carbosynth (Berkshire, United Kingdom). Naringenin and sodium carbonate, were purchased from VWR (Radnor, Penn., USA). Ampicillin and nystatin were purchased from Laboratoire Humeau (La Chapelle-sur-Erdre, France). Antibiotic cellulosic discs were purchased from Grosseron (Couëron, France).

*Prunus avium* var. *burlat* branches were collected from Chestnut (Montélimar (44°33′31″ N 04°45′03″ E), France). *Escherichia coli* (LMG 2092) and *Bacillus subtilis* (LMG 7135) were purchased from BCCM (Bruxelles, Belgium). *Candida albicans* (ATCC 22972) was purchased from DSMZ (Braunschweig, Germany).

Grinding was realized with SM300 knife mill (Retsch, Haan, NRW, Germany). Extractions were performed with Accelerated Solvent Extractor ASE 350 (Dionex Corporation, Sunnyvale, CA, USA). Extracts were evaporated on a 12 Position N-EVAP Nitrogen Evaporator (Organomation, Berlin, MA, USA). Spectrometric reads were carried out with Cary 60 Spectrophotometer (Agilent, Wilmington, DE, USA). High resolution mass spectrometry was performed on an Agilent 1290 system, equipped with a 6545 Q-TOF mass spectrometer (Wilmington, DE, USA), a Zorbax C18 column (2.1 mm × 50 mm, 1.8 µm) coupled with a PDA UV detector from Agilent (Santa Clara, CA, USA). The source was equipped with a Dual Agilent JetStream ESI probe operating at atmospheric pressure in positive or negative ionization mode. Pictures were taken with E-Box Vilver (Grosseron, Couëron, France) under UV (Focus: 1565, Zoom: ×2, Exposure: 320 ms). Lyophilization was performed with Labconco Freeze dryer (Kansas City, MO, USA).

### 2.1. Plant Material

*Prunus avium* var. *burlat* branches were ground with a 4 mm sieve, at 1500 min^−1^, during 1–2 min. Milled branches were kept at 4 °C until extraction.

### 2.2. Extraction Protocols

#### 2.2.1. Conventional Extraction Process

Conventional extraction by solvent was achieved to identify the PCs present in cherry branches. First, 825 mg of wet milled branches were introduced in a 250 mL flask with 36 mL of solvent (ethanol-water, 70/30, *v*/*v*) to achieve 0.05 mL/mg (solvent/dry matter (DM)). The flask was then covered with a flux condenser and maintained at 70 °C during 30 min under 350 rpm agitation. The crude was centrifugated at 4000× *g* and 25 °C during 10 min. Supernatant was recovered, filtered with a grade 11 filter, and kept at −20 °C.

#### 2.2.2. Accelerated Solvent Extraction Process

Experiments were carried out on using 34 mL stainless-steel cells, in static mode. The extraction cells were (i) filled with 825 mg of wet milled branches to achieve a ratio of 0.05 mL/mg_DM_; (ii) placed in the oven to reach desired temperature (from 25 to 150 °C); (iii) loaded with variable solvent composition (ethanol-water, 30 to 70%, *v*/*v*) at 1500 psi; (iv) and left in static mode for 30 min. Extracts were then collected in 60 mL vials thanks to a 30 s nitrogen purge and kept at −20 °C.

### 2.3. Identification and Quantification of PCs by UHPLC-MS

#### 2.3.1. Sample and Standard Preparation

Every extract was concentrated by a factor of five to facilitate the detection. Samples were evaporated with N-EVAP and solubilized with a 50/50 (*v*/*v*) Ethanol/ultra-pure water mixture, then stirred and filtered through a 0.2 μm RC filter.

Standards were dissolved in ethanol-water, 70/30, *v*/*v*. Appropriate concentration ranges (6 to 7 points) were achieved from commercial reference stock solutions to quantify extract samples.

#### 2.3.2. LC-MS Conditions

Column temperature was fixed at 40 °C and injection volume at 1 μL. Gradient elution solvent was set up as 0.1% (*v*/*v*) formic acid in water (solvent A) and 0.1% (*v*/*v*) formic acid in acetonitrile (solvent B) at a flow rate of 0.4 mL/min. Gradient was scheduled as follows: Solvent B; 0 to 3 min (5%), 3 to 5 min (5 to 10%), 5 to 7 min (10%), 7 to 8 min (10 to 14%), 8 to 10 min (14 to 18.6%), 10 to 12 min (18.6%), 12 to 19.5 min (18.6 to 36%), 19.5 to 23 min (36–50%), 23 to 25 min (50 to 65%), 25 to 30 min (65 to 100%), 30 to 32 min (100 to 5%), inspired by the method used by Fu et al. [15]. Absorbance spectra were recovered through diode array detector set at 210, 254, 285, and 320 nm.

Source parameters were fixed as follows for the positive mode: capillary voltage, 3500 V; nozzle voltage, 2000 V; fragmentor voltage, 175 V; skimmer 1, 65 V; octopole, 750 V; gas temperature, 325 °C; nitrogen gas flow rate, 8 L/min; sheath gas temperature, 350 °C; sheath gas flow rate, 11 L/min. Scan range was set between 50 and 1050 *m*/*z* and scan rate at 2 spectra per second. Reference ions were: 121.050873 and 922.009798 for the positive mode and 112.985587 and 1033.988109 for negative mode.

#### 2.3.3. Phenolic Compound Identification

All extracts were analyzed by LC-MS. The most intense peaks were extracted for each retention time. These results were then processed using MassHunter software version 8.0.0 (Agilent, Santa Clara, CA, USA). Data were firstly compared with a homemade database followed by a commercial library (NIST and Metlin databases) if no matches were obtained. Commercial standards of the main compounds were used to certify the first identifications. Spectra and retention times were compared to confirm the correct assignment of peaks.

### 2.4. Biological Assays

The antioxidant activity was assessed from the raw extracts obtained using ASE, whereas the antimicrobial and anti-tyrosinase activities were carried out from powder obtained after evaporation of the raw extracts.

#### 2.4.1. Antioxidant Activity

Antioxidant activity was assessed by the DPPH radical scavenging method standardized for (±)-6-Hydroxy-2,5,7,8-tetra-methylchromate-2-carboxylic acid (Trolox). The 2,2-diphenyl-1-picrylhydrazyl (DPPH) assay evaluates the ability of the sample to reduce the DPPH^+^ radical, by following the decolorization at 515 nm. 

DPPH solution and Trolox range at 46.7 mg/L and 20–150 µmol/L, respectively, were prepared in methanol. Next, 880 µL of DPPH solution were placed in tubes to read the initial absorbance (A_0_), and then 320 µL of diluted samples/Trolox range were added. The samples were kept at 25 °C for 30 min in the dark, and absorbances (A_f_) were read. Inhibition percentages were calculated for each sample/Trolox concentration using Equation (1):

(1) $$\mathrm{Percentage}\;\mathrm{of}\;\mathrm{inhibition}=\frac{\left(A_{0}-A_{f}\right)}{A_{0}}\times 100,$$

The dilutions of samples and trolox were adapted to obtain a percentage of inhibition between 20–80%.

Then, the slopes (a_i_) of the percentage of inhibition (%) versus total phenolic/Trolox concentration (mg/mL or mg_GAE_/mL) were determined for each sample and the trolox solution.

Finally, the results were expressed using the inhibitory concentration (IC) to reduce the initial DPPH^.+^ radical concentration to 50% (IC_50_ in µg/mL), calculated using the previous slopes.

(2) $${\mathrm{IC}}_{50,i}=\frac{\left(50\times 1000\right)}{a_{i}},\;$$

#### 2.4.2. Anti-Tyrosinase Activities

Tyrosinase inhibitor activity was measured by spectrophotometry based on the method described by Masamoto et al. [16]. First, 10 μL of inhibitor solution at different concentrations in DMSO were added to a 96-well microplate and mixed with ammonium formate buffer (60 μL, 50 mmol/L, pH 6.4). Then, 20 μL (0.8 mg/mL) of tyrosine in ammonium formate buffer were added. Finally, 10 μL of mushroom tyrosinase (5000 U/mL in ammonium formate buffer) were added and the assay mixture was then incubated at 37 °C for 10 min. After incubation, the amount of dopachrome production was determined at 450 nm in a microplate spectrophotometer. Kojic acid solutions, at different concentrations, were used as a positive control. The concentration needed for 50% of tyrosinase inhibition (IC_50_) was determined.

#### 2.4.3. Antimicrobial Activities

Agar plates were inoculated with a standardized bacterial or fungal suspension (10^6^ CFU/mL). Three different microorganisms were studied: *Escherichia coli*, *Bacillus subtilis*, and *Candida albicans*. The lyophilized extracts or standards were dissolved in a hydroalcoholic solution (40% ethanol) at different concentrations from 2.5% to 0.02% (*w*/*v*). The presence of ethanol in the samples deposited on the discs had no influence on the microorganism growth in the absence of active molecules. Ampicillin, ethyl paraben, phenoxyethanol, and nystatin were chosen as inhibition references to validate the tests. Next, 30 µL of tested samples were deposited on the surface of cellulose discs and placed on the inoculated agar plate. Then, the systems were incubated at 30 °C for a suitable period for the microorganism growth. The growth inhibition circle around the disc indicates the sensitivity of the microorganism to the extracts leading to qualitative results. The minimal concentration allowing to observe the inhibition circle is considered as minimum inhibitory concentration (MIC). 

### 2.5. Statistical Analysis

Outside of antimicrobial activities, each experiment and analysis were carried out as three replicates. Results were expressed as mean values ± standard deviations. Data were analyzed using freeware R 3.3.0. (Auckland, New Zealand). Analysis of Variance (ANOVA) was performed, followed by Tukey test. Differences with *p* < 0.05 were defined as significant. Letters are indicated for each series to indicate the groups formed by the Tukey test. Values in the same group are considered statistically equivalent.

## 3. Results

First, the PCs recovered from cherry branches were identified and quantified. Then, the effect of the ethanol percentage in the extraction solvent and the extraction temperature on the content of the identified PCs was studied. Finally, the influence of the operating conditions of the extraction process was assessed on the antioxidant, anti-tyrosinase and antimicrobial activities of the extracts.

### 3.1. Phenolic Compound Identification and Quantification

Extracts were obtained from cherry branches by conventional solvent extraction in ethanol-water, 70/30, *v*/*v,* at a temperature of 70 °C. The main PCs (14 molecules) were identified through databases and their presence was confirmed using commercial standards. The characteristics of the identified compounds obtained by mass spectrometry as well as their content are shown in Table 1.

Some compounds, having multiple *m*/*z*, were identified by fragmentation in the source. This is the case of prunin, a glucoside of naringenin, whose [M + H]^+^ is 435.1283 Da, and which, by losing a glucose, has a fragment at 273.0754 Da for the positive mode ionization. On the other hand, genistein and apigenin, which have the same raw formula and were not differentiated with the spectra of the extracts, could be differentiated thanks to the standards which have different retention times, 15.8 and 16.3 min, respectively.

**Figure 1:**
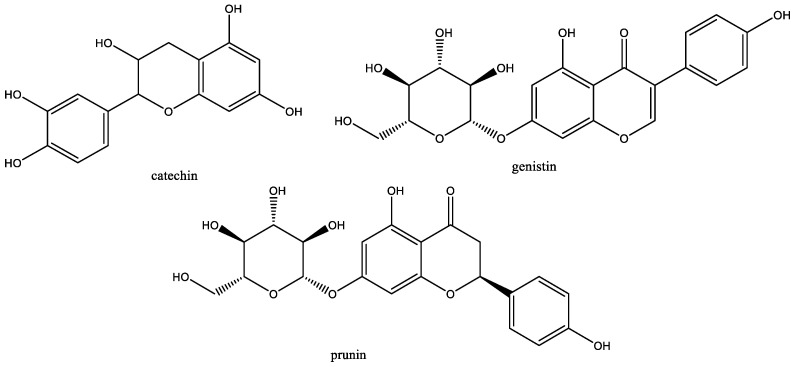
Chemical structures of the main PCs in cherry tree branch extracts.

Quantification of PCs in the extract was performed using calibration curves of standard solutions. The different contents are shown in Table 1. Three PCs have high contents representing between them 84% of the total PCs. These are –an isoflavone monoglycosylated (27.22 ± 1.01 mg/g_DM_), catechin–a flavan-3-ol (22.04 ± 0.53 mg/g_DM_), and prunin–a monoglycosylated flavonone (10.87 ± 0.38 mg/g_DM_). Chemical structures are grouped in Figure 1. 

Few studies have focused on identifying PCs from cherry branches. Most studies have determined PCs from cherry fruit, leaves, and stems. Nunes et al. reviewed the different PCs found in these three by-products [17]. Overall, 32 phenolic acids and 44 flavonoids were identified. All PCs mentioned in this paper were also found by other authors [11,18,19]. In particular, catechin, genistin, and prunin were found in the stems with the following contents: 5014.0–5259.5 µg/g_DM_ [19], 182 µg/g_DM_ [11], and 2836.4–4036.2 µg/g_DM_. PCs in the branches seem to be less numerous but with higher levels than in other cherry by-products.

### 3.2. Effect of the Extraction Process on the IPC Content of the Extracts

Two operating conditions were studied to optimize the IPC content; the ethanol percentage and the extraction temperature. For this, extractions were carried out using ASE in order to study temperatures higher than the ebullition point.

#### 3.2.1. Effect of the Percentage of Ethanol

**Figure 2:**
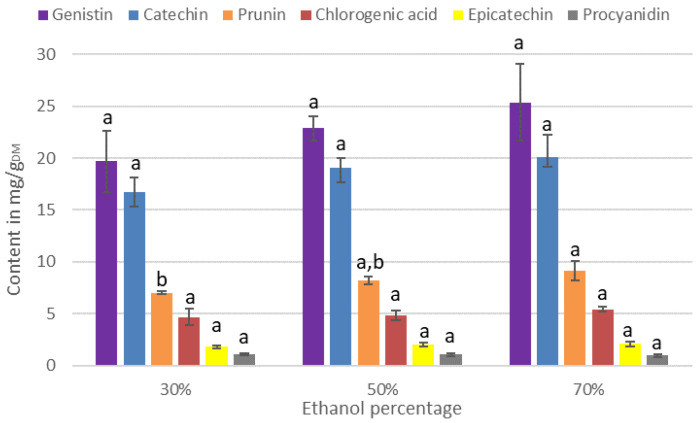
Contents of IPCs with different percentages of ethanol. For each series, the same letter indicates that no significant difference is found between the two means (Tukey’s test).

The contents of IPCs according to the ethanol percentage in the solvent of extraction are presented in Figure 2.

According to Figure 2, few PCs have their contents impacted by a change in the percentage of ethanol used during the extraction. Genistin, catechin, chlorogenic acid, epicatechin, procyanidin, genistein, neochlorogenic acid, naringenin, chrysin, and apigenin have a strong affinity with water as with ethanol, which explains the lack of effect of the percentage of ethanol on the extraction capacity. However, regarding prunin, quercetin, and rutin, their contents increased by 23%, 29%, and 34%, respectively, when 70% ethanol is used as extraction solvent instead of 30%. Thus, to maximize the content of total PCs in the extract, it is recommended to use 70% ethanol during extraction.

#### 3.2.2. Effect of the Temperature of Extraction

**Figure 3:**
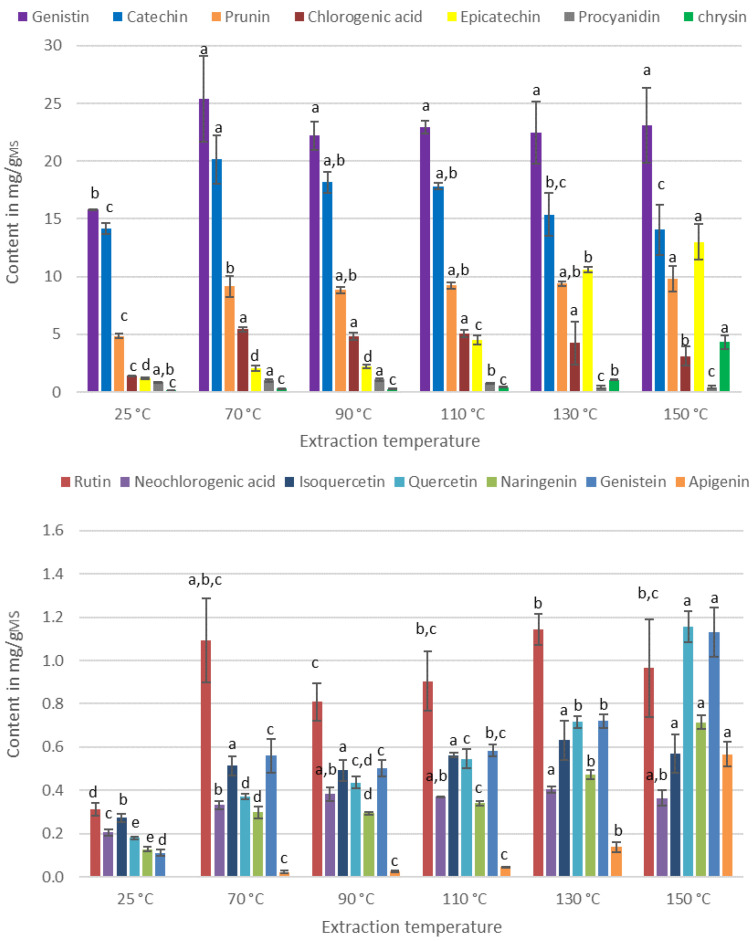
Contents of IPCs with different extraction temperatures. For each series, the same letter indicates that no significant difference is found between the two means (Tukey’s test).

Figure 3 presents the contents of IPCs for different extraction temperatures.

From Figure 3, the extraction temperature has a greater effect than the percentage of ethanol. An increase in the content of all molecules is observed between 25 °C and 70 °C. Indeed, the temperature has an impact on the solubility of the molecule in the solvent as well as on its diffusion. An increase in the temperature would thus improve the extraction capacity. However, the energy provided by higher temperature should not be too high since the PCs–being thermosensitive–can degrade, and therefore their contents decrease. 

The molecules behave differently under the effect of temperature. Concerning isoquercetin and genistin, the contents remain constant between 70 °C and 150 °C, while molecules such as rutin, procyanidin, and catechin have their optimum at 70 °C. Higher temperatures lead to a decrease in their contents, certainly due to the degradation of the molecules. Chlorogenic and neochlorogenic acids have a similar content between 70 °C and 130 °C, but a decrease is observed for temperatures above 130 °C. Conversely, quercetin, naringenin, genistein, apigenin, epicatechin, and chrysin have their contents increase gradually to reach the highest value at a temperature of 150 °C.

In the literature, it has been shown that model solutions of glycosylated flavonoids are more stable to heat treatment than those of aglycon flavonoids, due to their higher activation energy [20]. Our results are not in agreement with this observation. Indeed, quercetin shows its highest content at 150 °C, while rutin (quercetin diglycoside) is degraded at this temperature. This can be explained by several phenomena: (i) the molecules being in a matrix, the latter could influence their sensitivity. Indeed, Murakami et al. found that the presence of chlorogenic acid has a protective effect on rutin against thermal degradation [21]; (ii) the absence of oxygen due to the use of ASE for extraction would modify the sensitivity of PCs to temperature. Indeed, it has been shown that the oxidative degradation of molecules by high temperatures is stronger with higher oxygen content in the medium [22,23]; (iii) the duration of the heat treatment may be too short to observe a significant decrease. From a 2012 review, it was concluded that the degradation of flavonoid solutions can be considered proportional to the time–temperature combination [24]. For example, a model rutin solution is completely degraded after 30 min at a temperature of 130 °C while, at 90 °C, only 15% is degraded for the same duration of heat treatment [20]. 

**Figure 4:**
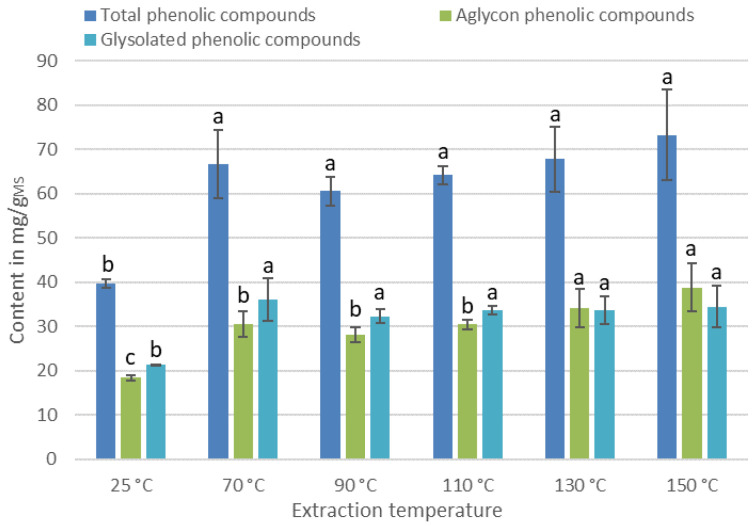
Evolution of the content of total aglycons and glycosylated PCs. For each series, the same letter indicates that no significant difference is found between the two means (Tukey’s test).

The proportion of the different PCs in the extract being different, it is relevant to observe the content evolution of total, aglycones, and glycosylated PCs as a function of temperature (Figure 4).

For total and glycosylated PCs, a temperature of 70 °C is necessary and sufficient to obtain maximum content. On the other hand, to optimize the content of aglycone PCs, it is necessary to apply at least a temperature of 130 °C. Thus, the extraction temperature could be a parameter with which to influence the selectivity of the extraction process. A high extraction temperature leads to an extract rich in aglycone PCs, which could provide the extract with different biological activities. 

### 3.3. Effect of the Process on Biological Activities of the Extracts

Several biological activities are particularly sought after for cosmetic products. Antioxidant activity is essential to protect the skin from external aggressions, but also to protect and stabilize the formulations and their active ingredients. The safety of the formulations is also important, so antimicrobial activities are sought to replace several controversial preservatives. Finally, anti-tyrosinase activity–which results into a skin whitening effect–is a biological activity that represents an important part of the cosmetic market. The study of the influence of the extraction conditions on the biological activities of the extracts will then allow to optimize the conditions for a targeted application.

#### 3.3.1. Antioxidant Activity Measured by DPPH

**Figure 5:**
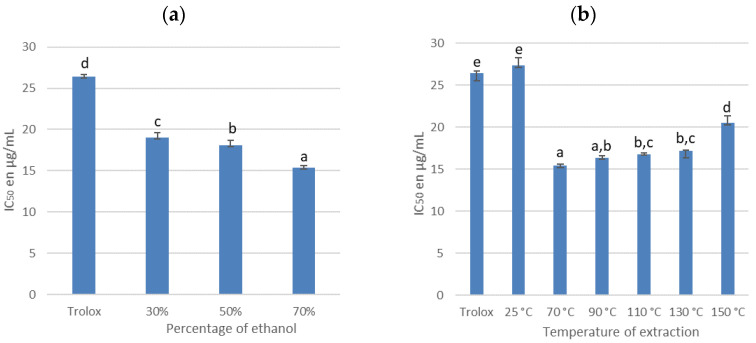
IC_50_ for different percentages of ethanol (**a**) and for different extraction temperature (**b**). For each series, the same letter indicates that no significant difference is found between the two means (Tukey’s test).

Figure 5 presents the IC_50_ of the extracts according the operating conditions of the extraction process.

The most antioxidant extracts are characterized by a low IC_50_. Our reference is trolox, for which we obtained an IC_50_ of 26.40 ± 0.32 µg/mL. The extracts obtained from cherry branches are more antioxidant than the trolox whatever the operating conditions of the extraction process. However, significant differences are noticed according to the percentage of ethanol and the extraction temperature. The most favorable extraction conditions for high antioxidant activity are 70% ethanol, at 70 °C. Under these conditions, the extract shows an IC_50_ of 15.39 ± 0.19 µg/mL.

**Figure 6:**
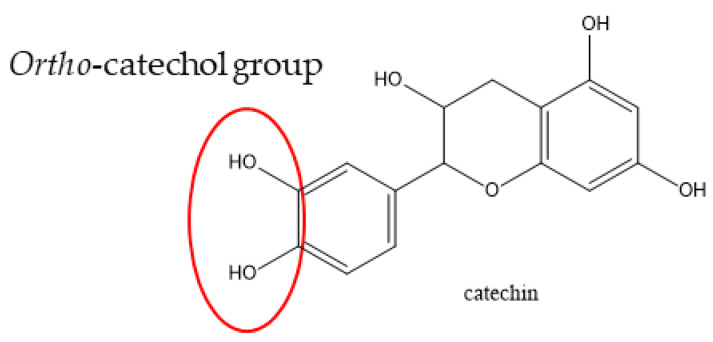
Structure of the catechin.

The effect of the operating conditions on the IC_50_ could be explained by variations of the IPC contents in the extract. Concerning the ethanol percentage, we observed in Figure 2 that only the contents of rutin, prunin, and quercetin are significantly higher with 70% ethanol. Thus, the increase in antioxidant activity when the extract is produced with 70% ethanol can be explained either by the presence of these three molecules, or by the existence of a synergy between them and the other PCs. Concerning the extraction temperature, only the catechin and chlorogenic acid contents are significantly affected by an increase in the extraction temperature. Catechin is known to be a powerful antioxidant due to its *ortho*-catechol group (3′,4′-OH) and the presence of four phenoxy groups (Figure 6) [25]. Moreover, its content in the extract is very high, corresponding to 44% of the PCs in the extract. Thus, the optimal antioxidant activity obtained for an extraction at a temperature of 70 °C is certainly related to the catechin content.

#### 3.3.2. Anti-Tyrosinase Activity

**Figure 7:**
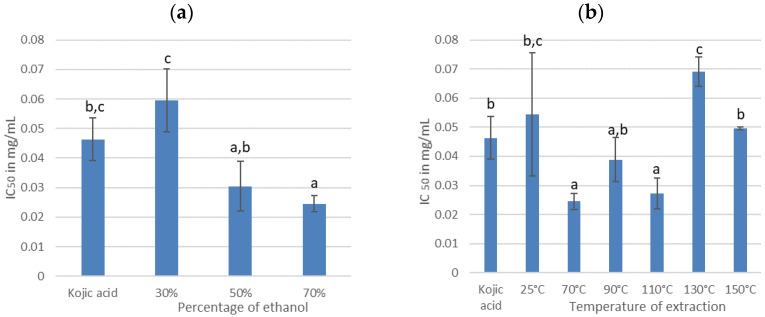
IC_50_ for different percentages of ethanol (**a**) and for different extraction temperature (**b**). For each series, the same letter indicates that no significant difference is found between the two means (Tukey’s test).

Tyrosinase is the key enzyme in the melanin biosynthesis, the main brown pigment of the skin. Aging or intense sun exposure can lead to the appearance of hyper pigmentation disorders–brown spots that can be controlled by limiting the production of melanin–thanks to the reduction in tyrosinase activity. Thus, plant extracts able to reduce tyrosinase activity represents a great potential for cosmetic application. As for the antioxidant activity, the effect of extraction conditions on the ability of extracts to inhibit tyrosinase was investigated. The results obtained are gathered in Figure 7. 

IC_50_ values were determined for each extract. The lower the IC_50_ value, the better the anti-tyrosinase activity. These values were then compared with that of kojic acid (0.046 ± 0.007 mg/mL), which is used as a positive reference. The extracts obtained from cherry branches present IC_50_ ranging from 0.025 to 0.069 mg/mL depending on the extraction process conditions. 

According to Figure 7a, the extract obtained with 70% ethanol exhibits the highest anti-tyrosinase activity (0.025 ± 0.003 mg/mL). It is more active than our reference, kojic acid. The 70% ethanol percentage has already been reported previously as a condition maximizing total and glycosylated PCs as well as antioxidant activity. Concerning the IPC composition, prunin, rutin, and quercetin are favored using 70% ethanol while catechin showed a moderate effect as a tyrosinase inhibitor, no information, to the best of our knowledge, was found for rutin and prunin [26].

According to Figure 7b, the extraction temperature has also a significant effect on the antioxidant activity of the extracts. The most active were obtained between 70 °C (0.025 ± 0.003 mg/mL) and 110 °C (0.027 ±0.005 mg/mL). These results can be explained by the variation in catechin content. As shown in Figure 4, the catechin content increases by 43% when the temperature rises from 25 °C to 70 °C and is strongly degraded for temperatures above 130 °C.

The most competitive IC_50_ was obtained with 70% ethanol and a temperature of 70 °C; moreover, in these conditions, the value was lowest than kojic acid. It means that the extracts of cherry tree branches could be a perfect alternative to kojic acid as whitening agent.

#### 3.3.3. Antimicrobial Activity

The cosmetic industry is looking for new natural alternatives to the use of synthetic preservatives (paraben, phenoxyethanol). Extracts obtained from biomass seems interesting if they possess a broad spectrum of action and can target several microorganisms at the same time. In order to evaluate the potential of cherry tree extracts, we determined their antimicrobial activity on three types of microorganisms: a Gram + bacterium (*Bacillus subtilis*), a Gram—bacterium (*Escherichia coli*), and a yeast (*Candida albicans*). The antimicrobial activities were evaluated by the capacity of the extracts to inhibit the growth of the microorganisms. Based on the principle of antibiograms, discs were soaked with different extracts in the presence of microorganisms to observe or not the appearance of an inhibition halo and to deduce the minimum inhibitory concentration (MIC). The results are summarized in Table 2 and Supplementary materials.

Four well-known preservatives were chosen as references. Ampicilline, which is an antifungal, showed inhibition of yeast with the presence of a 26 mm circle. Nystatin, which is an antibacterial, inhibited the growth of *E. coli* and *B. subtilis* with circles of 22 mm. Concerning phenoxyethanol and ethyl paraben, these molecules are often present in cosmetic formulations [27,28]. Phenoxyethanol is used to inhibit *E. coli* and *B. subtilis*, whereas ethyl paraben inhibits the two bacteria and *C. albicans*; the inhibition circles are 12 mm with a CMI of 1.25%. Looking at the results in Table 2, we noticed that none of the extracts produced has a spectrum of action as wide as that of ethyl paraben.

Concerning *E. coli*, only the extracts obtained at a temperature superior to 110 °C are able to inhibit its growth with a MIC between 1.25% and 2.5%. An antifungal effect (MIC of 2.5%) on *C. albicans* was obtained only with the extract obtained at 150 °C. For *B. Subtilis*, only the extracts obtained at 70 °C with 70% ethanol allowed its inhibition (MIC of 1.25%).

From these observations, we can conclude that extracts obtained at high extraction temperatures–previously qualified as rich in aglycone PCs–seem to inhibit yeasts such as *C. albicans* and Gram- like *E. coli*. Chrysin, whose content in the extract increased from 0.29 ± 0.03 mg/g_DM_ (70 °C) to 4.31 ± 0.56 mg/g_DM_ (150 °C), is known to have antifungal activity [29]. On the contrary, extracts obtained at moderate temperature (70 °C/70% ethanol), rich in catechin, prunin, and rutin, would have the capacity to inhibit Gram + such as *B. Subtilis*. These results are in agreement with the literature. Indeed, Fathima et al. have highlighted the antibacterial activity of catechin, which causes membrane permeabilization by oxidative damage [30].

## 4. Conclusions

Cherry tree branches are rich in PCs, mainly catechin (22.04 *±* 0.53 mg/g_MS_), genistin (27.22 *±* 1.01 mg/g_MS_), and prunin (10.87 ± 0.38 mg/g_MS_). Different extraction conditions were tested to compare the PC composition and the bioactivities of the different extracts. The extraction temperature has a greater effect than the ethanol percentage on the PC contents. An extract enriched in aglycon compounds is obtained by high extraction temperatures (above 110 °C) whereas glycosylated compounds are favored by an extraction temperature of 70 °C. Variations in antioxidant and anti-tyrosinase activities seem to be correlated to the catechin content. The antimicrobial activity is higher for extracts obtained at high temperature (150 °C). The most polyvalent extracts for cosmetic applications are those obtained with 70% ethanol at a temperature of 70 °C.

## Figures and Tables

**Table 1:** Characteristics of PCs identified (retention time, UV detection, masses and content).

PC	Retention Time (min)	UV Detection (nm)	Observed *m*/*z* (Da)	Content in mg/g_DM_
Neochlorogenic acid	1.5	320	355.1021/163.0387	0.42 ± 0.02
Catechin	3.0	285	291.0867/139.0387	22.04 ± 0.53
Chlorogenic acid	3.3	320	355.1029/163.0390	3.81 ± 0.32
Procyanidine B2	5.4	285	579.149	1.30 ± 0.05
Epicatechin	6.1	285	291.0867/139.0389	2.56 ± 0.05
Rutin	10.1	254	611.1616/303.0495/465.1026	1.19 ± 0.06
Genistin	10.2	254	433.1140/271.0599	27.22 ± 1.01
Isoquercetin	10.2	254	465.1035/303.0497	0.57 ± 0.02
Prunin	11.1	285	435.1283/273.0754/153.0182	10.87 ± 0.38
Quercetin	14.2	254	303.0509	0.41 ± 0.04
Naringenin	15.6	285	273.0756	0.33 ± 0.05
Genistein	15.8	254	271.0598	0.47 ± 0.03
Apigenin	16.3	320	271.0605	0.02 ± 0.01
Chrysin	20.4	254	255.0654	0.31 ± 0.01

**Table 2:** Assessment of antimicrobial activity according to the extraction conditions.

Factor Tested	Temperature (°C)	Ethanol Percentage	MIC		
			*E. coli*	*C. albicans*	*B. subtilis*
References	Ampicilline		10 µg (+22 mm)	No inhibition	10 µg (+22 mm)
	Nystatine		No inhibition	100 UI (+26 mm)	No inhibition
	Phenoxyethanol		1.25%	No inhibition	1.25%
	Ethyl paraben		1.25%	1.25%	1.25%
Variation of ethanol percentage	70 °C	30%	No inhibition	No inhibition	No inhibition
	70 °C	50%	No inhibition	No inhibition	No inhibition
	70 °C	70%	No inhibition	No inhibition	No inhibition
Variation of extraction temperature	25 °C	70%	No inhibition	No inhibition	1.25%
	70 °C	70%	No inhibition	No inhibition	No inhibition
	90 °C	70%	No inhibition	No inhibition	1.25%
	110 °C	70%	2.5%	No inhibition	No inhibition
	130 °C	70%	1.25%	No inhibition	No inhibition
	150 °C	70%	1.25%	2.5%	No inhibition

## Data Availability

Data is contained within the article and Supplementary Materials.

## Supplementary Materials

The following are available online at https://www.mdpi.com/article/10.3390/antiox11050813/s1. Figure S1: Calibration curves for the anti-bacterial activity; Figure S2: Pictures of the antimicrobial test results.
