# Supplementary material for: Phenolic Compounds Extracted from Cherry Tree (Prunus avium) Branches: Impact of the Process on Cosmetic Properties
Source: Antioxidants (Basel). 2022 Apr 22;11(5):813. doi: 10.3390/antiox11050813 (PMC9138022; doi:10.3390/antiox11050813)
Supplement: Supplementary file 1 [file antioxidants-11-00813-s001.zip › antioxidants-1664523-supplementary.pdf]

Supplementary Information

Phenolic Compounds Extracted from Cherry Tree (*Prunus avium)* Branches: Impact of the Process on Cosmetic Properties

Gaëlle Willig [1], Fanny Brunissen [1], Fanny Brunois [1], Blandine Godon [1], Christian Magro [2], Charles Monteux [2], Cédric Peyrot [1,*] and Irina Ioannou [1,*]

[1] URD Agro-Biotechnologies Industrielles (ABI), CEBB, AgroParisTech, 51110 Pomacle, France
[2] Chestnut, 26 Rue Barthélémy de Laffemas, 26000 Valence, France

Table of Contents

1. Calibration curves for the anti-bacterial activity S3
2. Antimicrobial tests according to the ethanol percentage S4
   2.1. Extraction with 30 % ethanol at 70°C S4
      2.1.1. E.coli S4
      2.1.2. C.albicans S4
      2.1.3. B.subtilis S4
   2.2. Extraction with 50 % ethanol at 70°C S4
      2.2.1. E.coli S4
      2.2.2. C.albicans S5
      2.2.3. B.subtilis S5
   2.3. Extraction with 70 % ethanol at 70°C S5
      2.3.1. E.coli S5
      2.3.2. C.albicans S5
      2.3.3. B.subtilis S5
3. Antimicrobial tests according to the extraction temperature S6
   3.1. Extraction with 70 % ethanol at 25°C S6
      3.1.1. E.coli S6
      3.1.2. C.albicans S6
      3.1.3. B.subtilis S6
   3.2. Extraction with 70 % ethanol at 70°C S6
      3.2.1. E.coli S6
      3.2.2. C.albicans S7
      3.2.3. B.subtilis S7
   3.3. Extraction with 70 % ethanol at 90 °C S7
      3.3.1. E.coli S7
      3.3.2. C.albicans S7

3.3.3. B.subtilis S7
3.4. Extraction with 70 % ethanol at 110°C S8
3.4.1. E.coli S8
3.4.2. C.albicans S8
3.4.3. B.subtilis S8
3.5. Extraction with 70 % ethanol at 130°C S8
3.5.1. E.coli S8
3.5.2. C.albicans S8
3.5.3. B.subtilis S9
3.6. Extraction with 70 % ethanol at 150°C S9
3.6.1. E.coli S9
3.6.2. C.albicans S9
3.6.3. B.subtilis S9

1. Calibration curves for the anti-bacterial activity

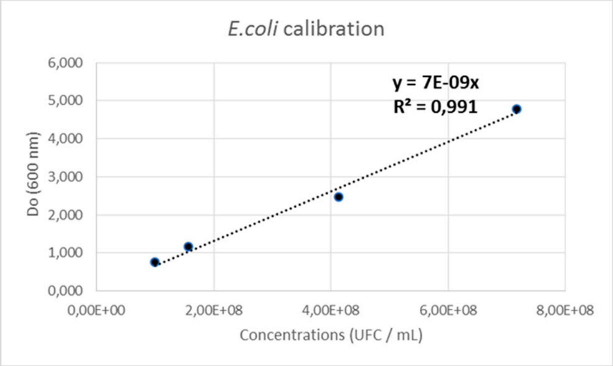

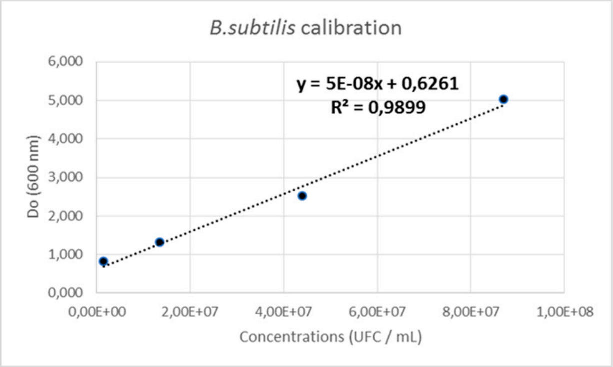

Figure S1: Calibration curves for the anti-bacterial activity.

2. Antimicrobial tests according to the ethanol percentage

2.1. Extraction with 30 % ethanol at 70°C

2.1.1. E.coli

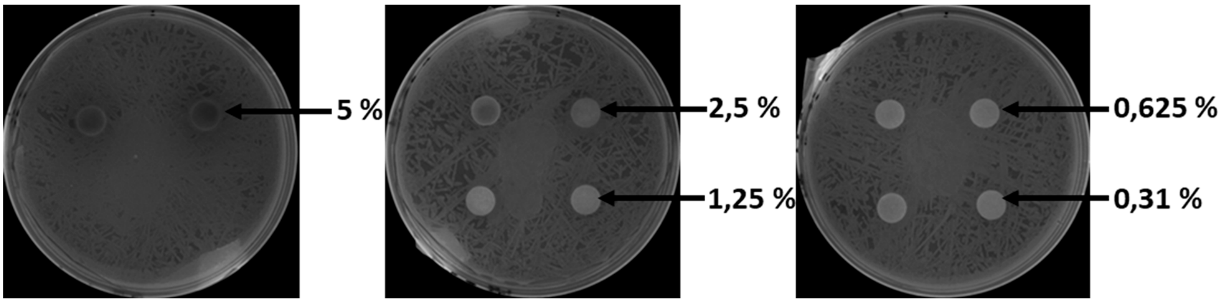

2.1.2. C.albicans

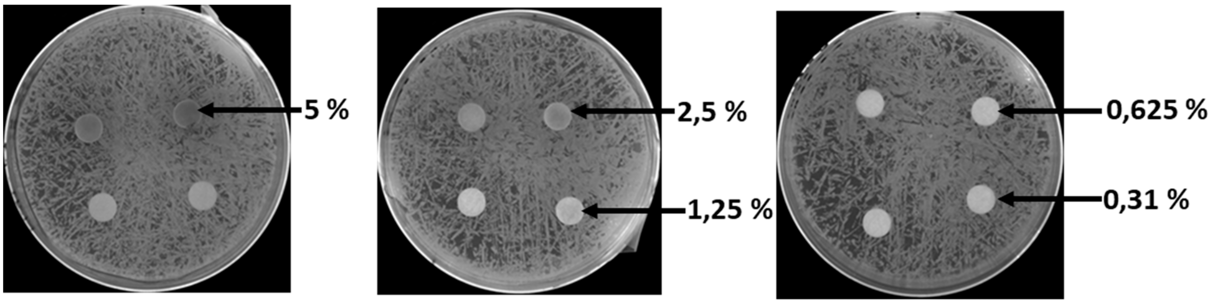

2.1.3. B.subtilis

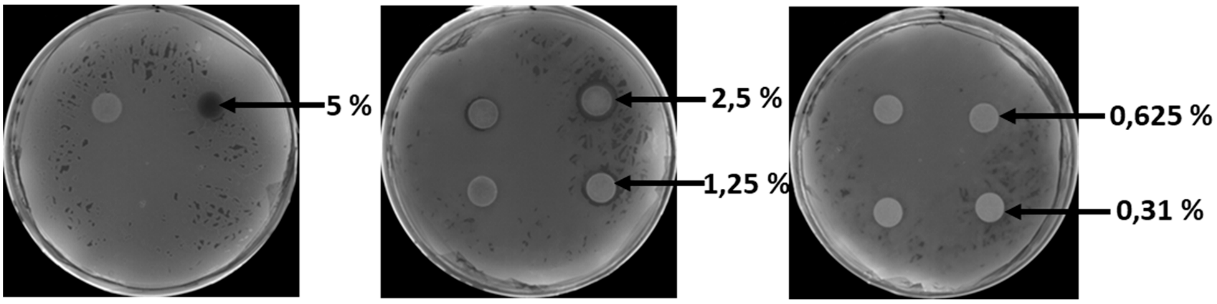

2.2. Extraction with 50 % ethanol at 70°C

2.2.1. E.coli

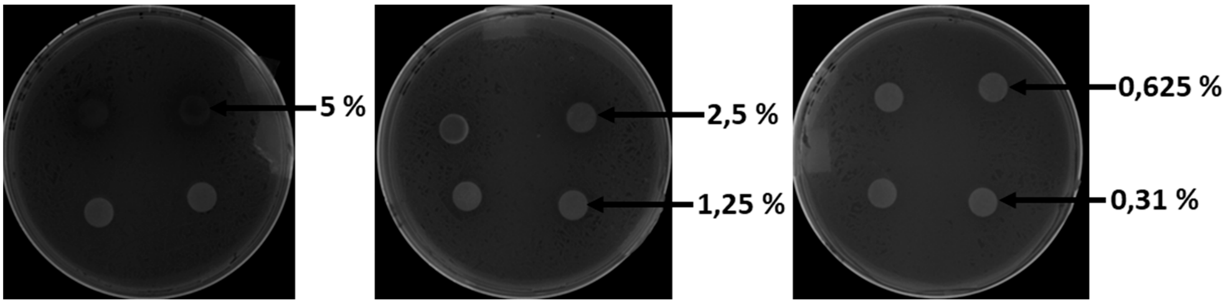

2.2.2. C.albicans

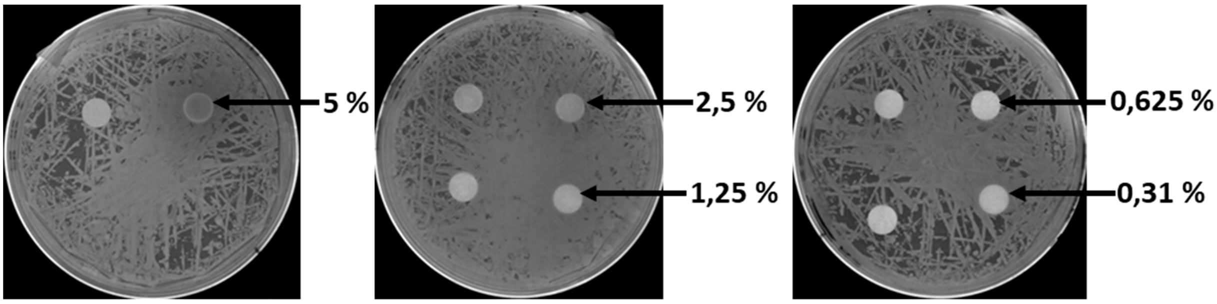

2.2.3. B.subtilis

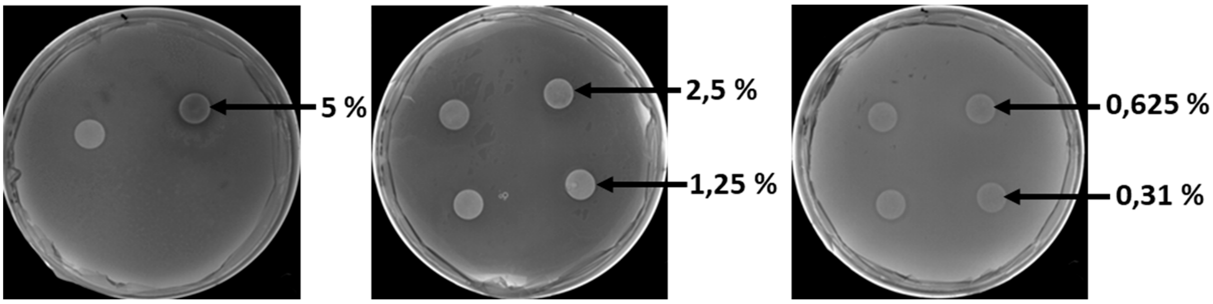

2.3. Extraction with 70 % ethanol at 70°C

2.3.1. E.coli

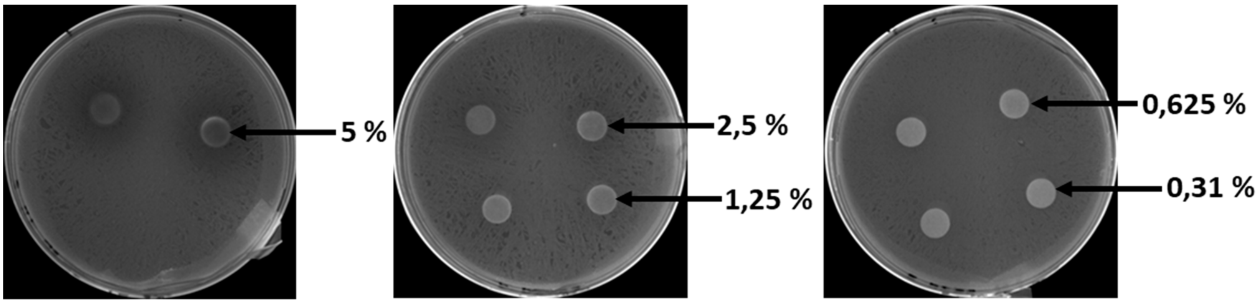

2.3.2. C.albicans

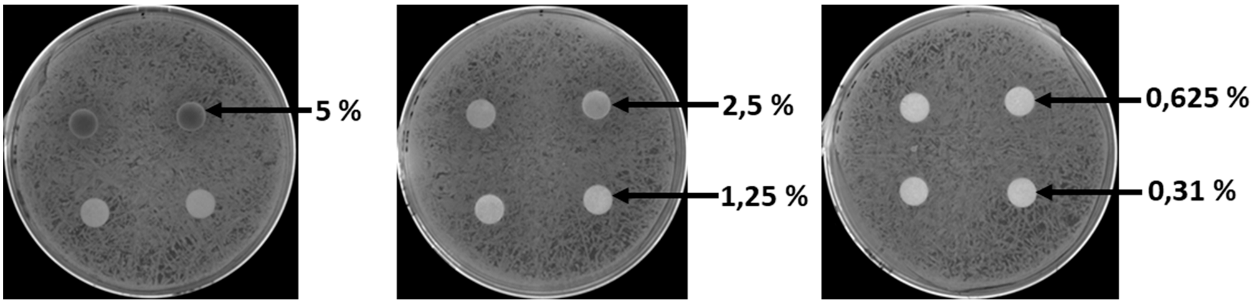

2.3.3. B.subtilis

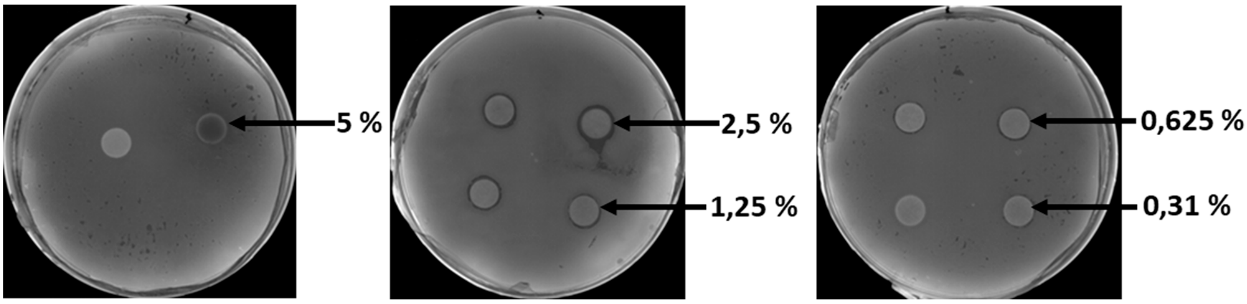

3. Antimicrobial tests according to the extraction temperature (Figure S2)

3.1. Extraction with 70 % ethanol at 25°C

3.1.1. E.coli

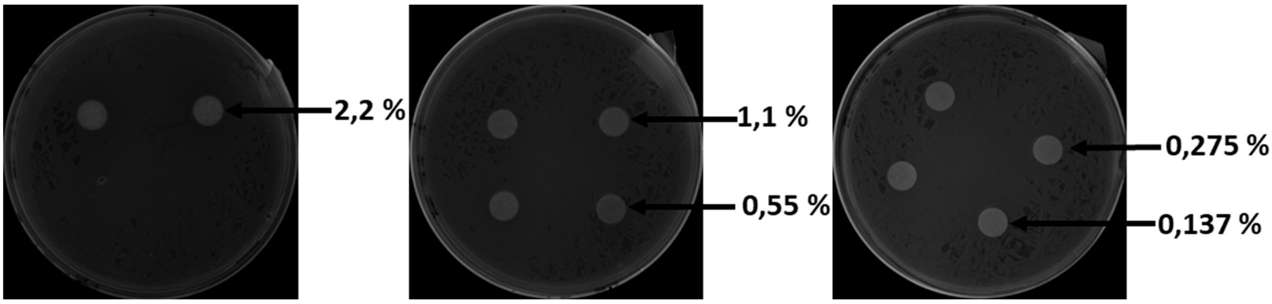

3.1.2. C.albicans

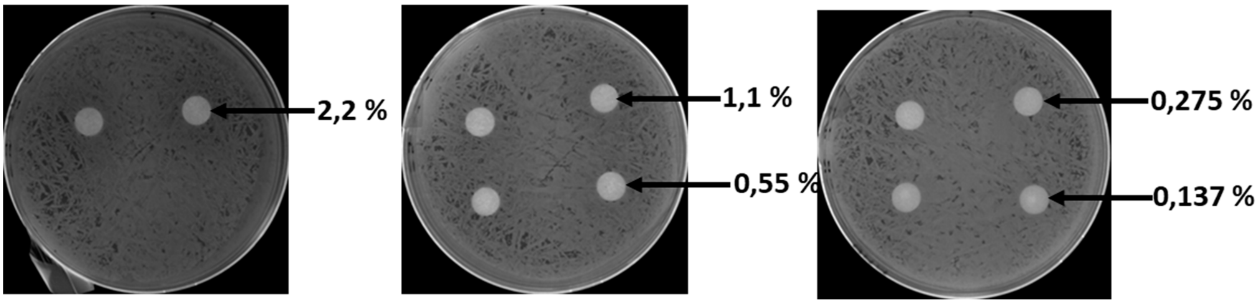

3.1.3. B.subtilis

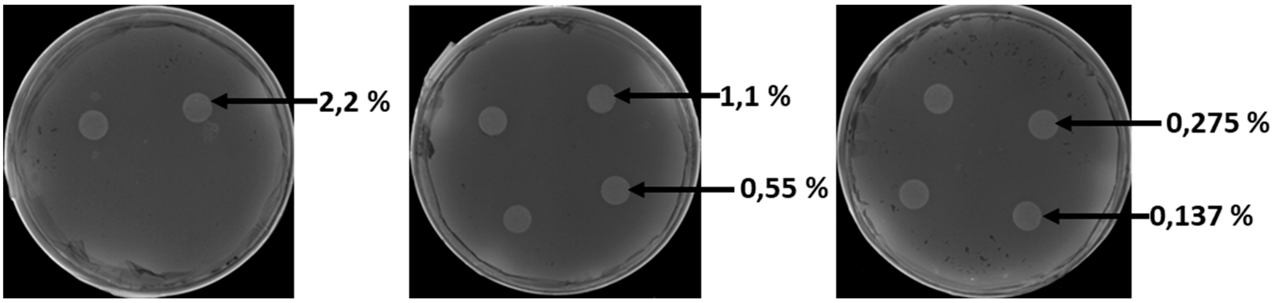

3.2. Extraction with 70 % ethanol at 70°C

3.2.1. E.coli

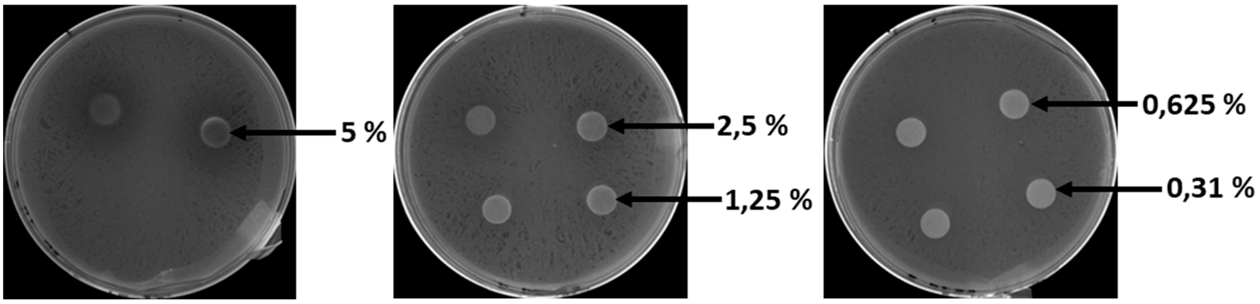

3.2.2. C.albicans

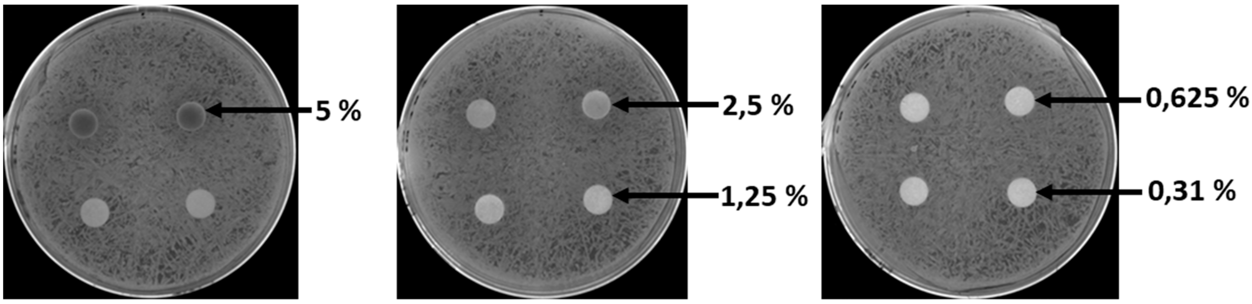

3.2.3. B.subtilis

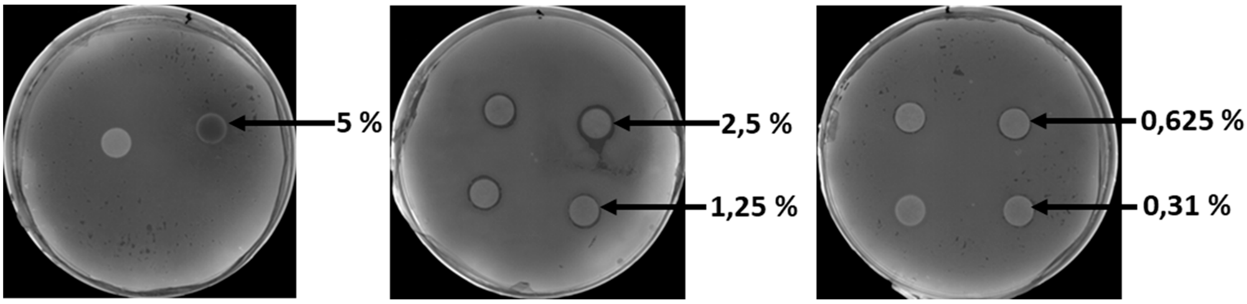

3.3. Extraction with 70 % ethanol at 90 °C

3.3.1. E.coli

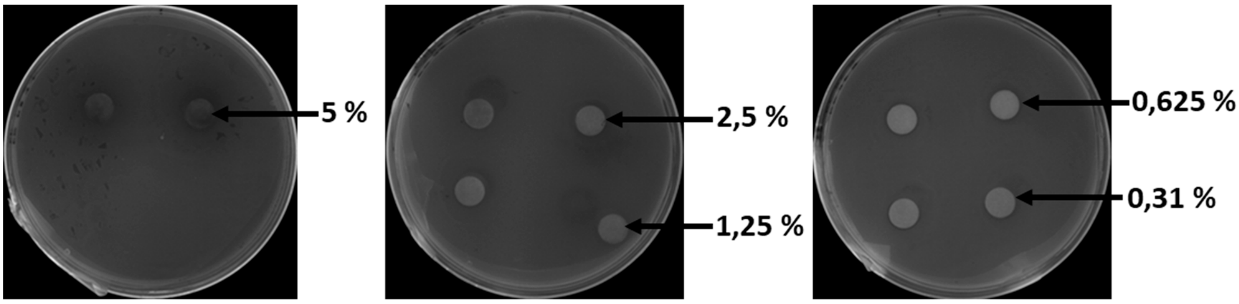

3.3.2. C.albicans

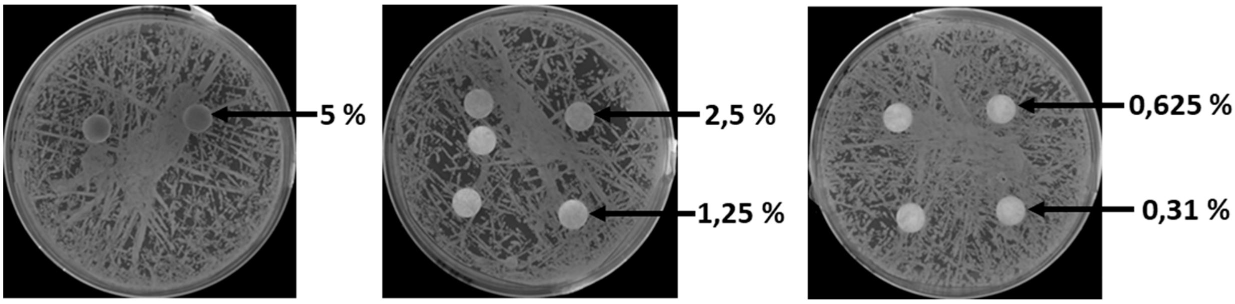

3.3.3. B.subtilis

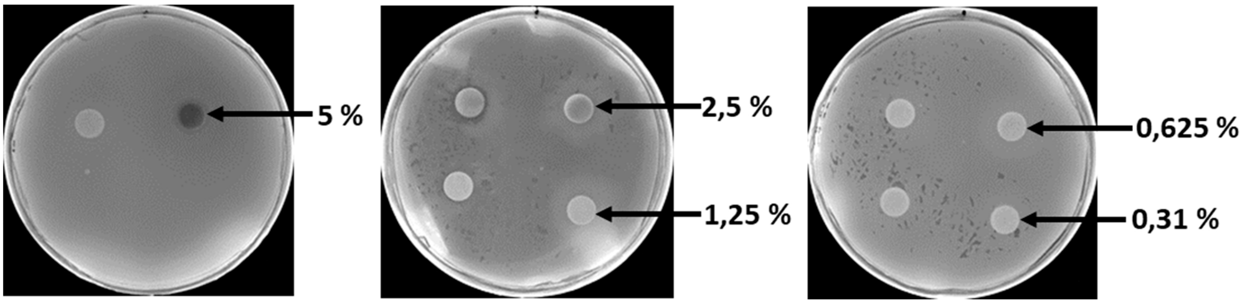

3.4. Extraction with 70 % ethanol at 110°C

3.4.1. E.coli

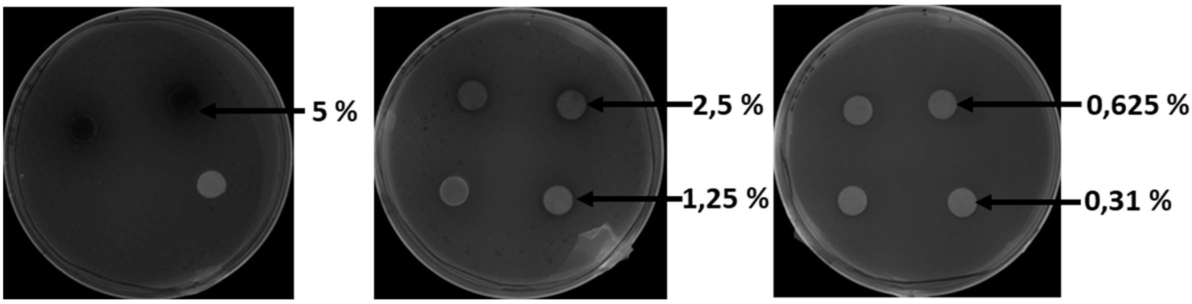

3.4.2. C.albicans

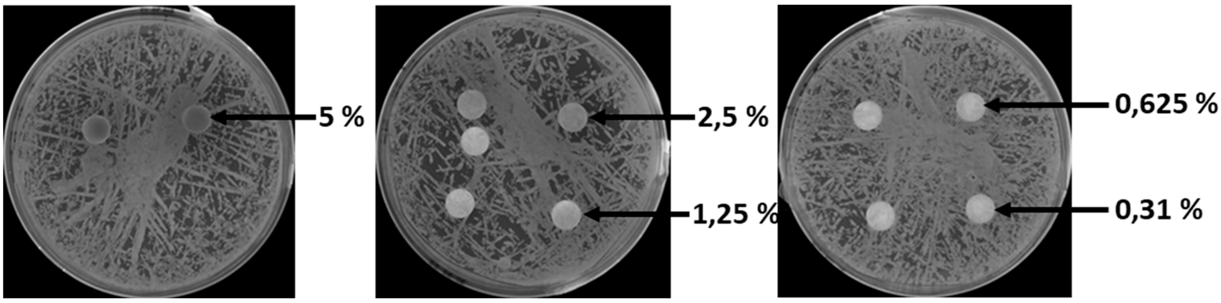

3.4.3. B.subtilis

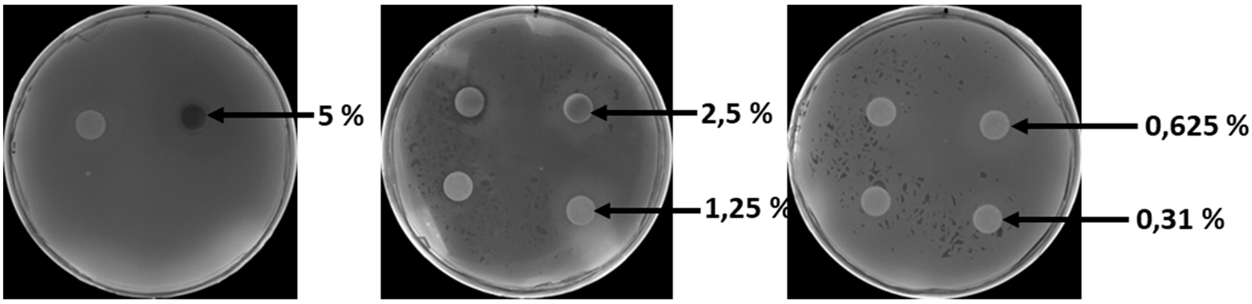

3.5. Extraction with 70 % ethanol at 130°C

3.5.1. E.coli

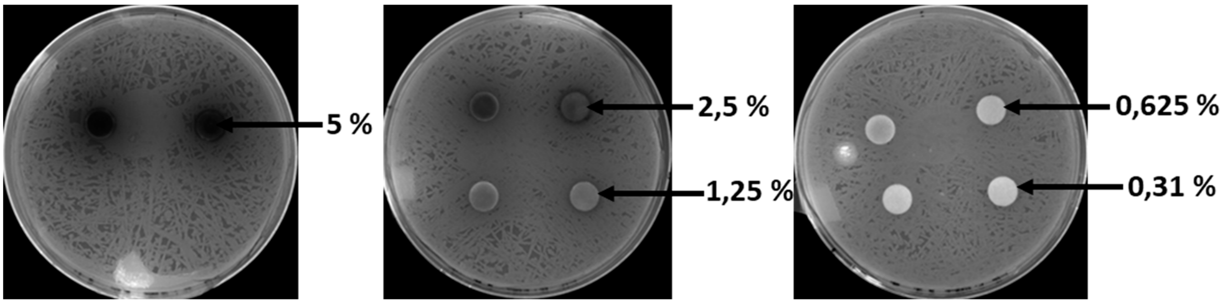

3.5.2. C.albicans

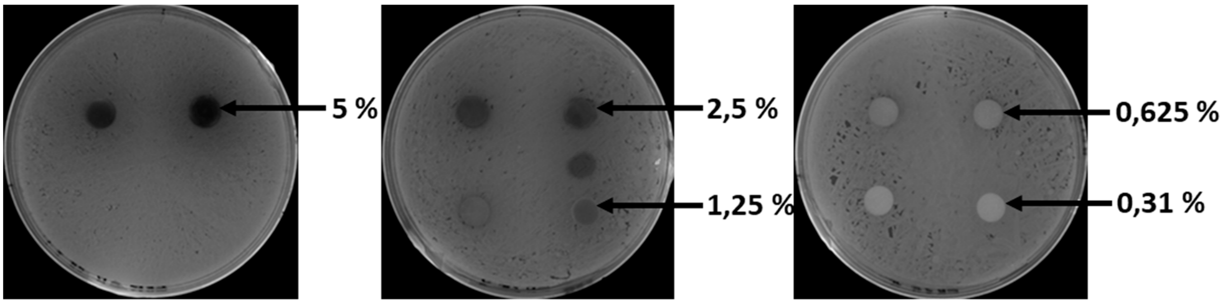

3.5.3. B.subtilis

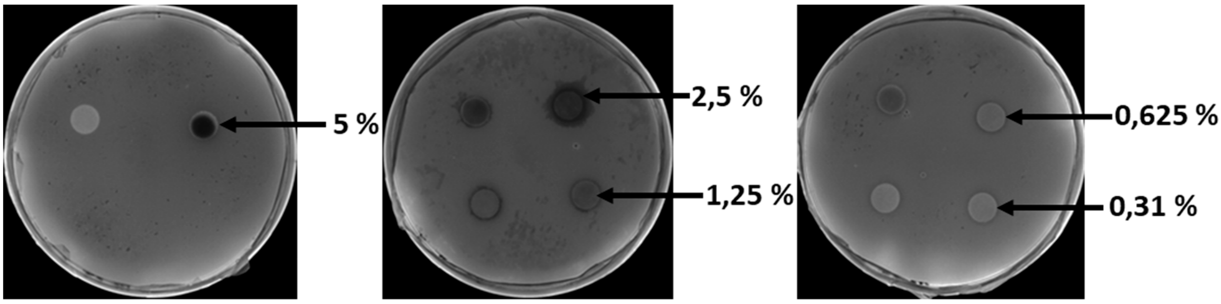

3.6. Extraction with 70 % ethanol at 150°C

3.6.1. E.coli

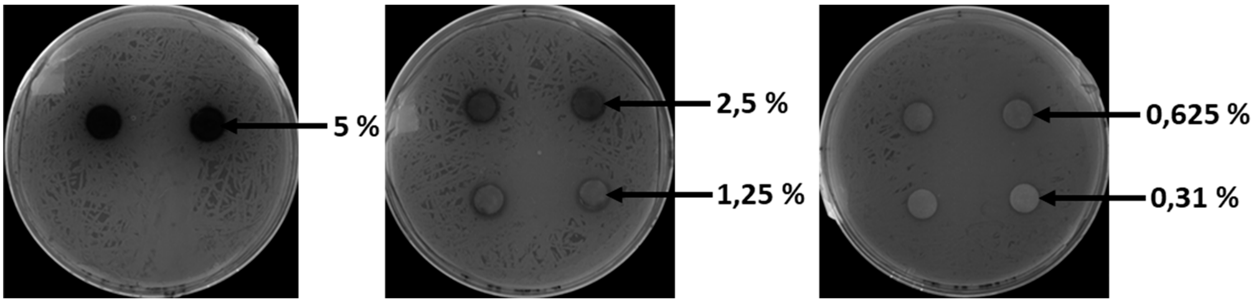

3.6.2. C.albicans

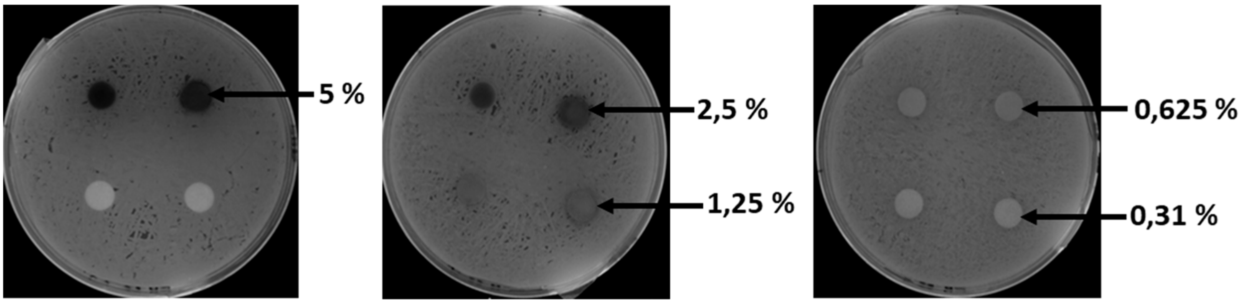

3.6.3. B.subtilis

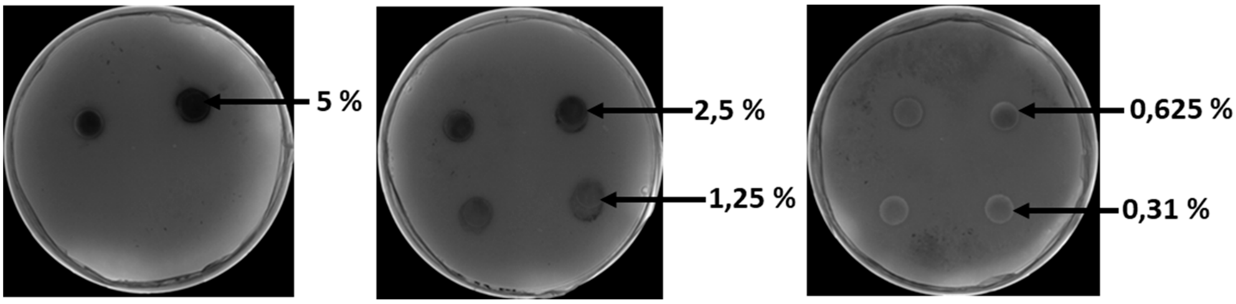

Figure S2: Pictures of the antimicrobial test results.
